# Assessing Awareness and Knowledge of Breast and Cervical Cancer Among Appalachian Women

**Published:** 2006-09-15

**Authors:** Nikki L Lyttle, Kelly Stadelman

**Affiliations:** West Virginia Department of Health and Human Resources, West Virginia Breast and Cervical Cancer Screening Program; RMS Strategies, Inc, Charleston, WVa

## Abstract

**Introduction:**

West Virginia is the only state that lies entirely within Appalachia. West Virginians tend to be poorer and more likely to lack health insurance than the general U.S. population. The purpose of this qualitative study was to 1) obtain an understanding of attitudes about breast and cervical cancer screening among women aged 25 to 64 years; 2) determine factors that motivate women to be screened for breast and cervical cancer; and 3) evaluate educational materials about breast and cervical cancer screening for use in this population.

**Methods:**

The West Virginia Breast and Cervical Cancer Screening Program (WVBCCSP) is a comprehensive public health program, funded by the Centers for Disease Control and Prevention, dedicated to removing barriers to breast and cervical cancer screening and providing screenings to underserved women aged 25 to 64 years. The program partnered with RMS Strategies, Inc, to conduct six focus groups in three communities in West Virginia. Women were recruited by telephone based on program eligibility guidelines.

**Results:**

Results indicated that women were concerned about health care costs and lack of health insurance. Cost, fear, and embarrassment were identified as the top barriers to breast and cervical cancer screening. Participants believed that community-based educational campaigns would increase screening and promote use of the WVBCCSP.

**Conclusion:**

Understanding why low-income Appalachian women do not get screened for breast and cervical cancer and determining motivational factors that encourage screening are important to increase screening rates among this population. Breast and cervical cancer efforts that use the words, knowledge, and suggestions of the women they serve are more likely to be effective and have a larger impact.

## Introduction

West Virginia is the only state in which all of the counties lie entirely within the Appalachian region. The region, as well as West Virginia, is largely rural and demographically homogeneous. West Virginia's population is 95% white, compared with 92% for Appalachia (1,2). Approximately 42% of Appalachians reside in rural areas, compared with 20% of the U.S. population (3). Additionally, both West Virginia and the Appalachian region as a whole are characterized by high poverty rates, low educational levels, and an aging population (4).

In 2000, the median age of West Virginians was 38.9 years, 3.6 years older than the national median (1). According to the U.S. Census Bureau's 2004 American Community Survey Data Profile Highlights, the median age of West Virginians was 40.3, up 1.4 years from 2000 and more than 4 years higher than the national median (5).

West Virginia ranks 48th among all U.S. states and the District of Columbia for high school graduates and 51st for college graduates (5). Only 79.4% of residents aged 25 years and older have a high school diploma or high school equivalency, compared with 83.9% nationally, and only 16.3% have a college degree, compared with 27.0% nationally (5). West Virginians tend to be poorer than their national counterparts; 17.9% of individuals live in poverty, placing West Virginia fifth in the nation for residents living below the poverty level (5).

Cancer mortality is higher in Appalachia than the rest of the United States, with rural Appalachia being affected the most. Several small studies identified age, lower income or education level, or lack of health insurance as factors contributing to low rates of breast and cervical cancer screening among Appalachian women (6). Approximately 13% of Appalachians are considered to be medically indigent (4). The rate of mammography among Appalachian women was 3.2% lower than the national rate, and the Papanicolaou (Pap) test rate was 2.5% lower (6).

According to the American Cancer Society, three in five (60.5%) women aged 40 years and older in West Virginia had a mammogram within the past year. Women aged 65 and older are slightly less likely to have had a mammogram in the past year (57.9%) than women aged 40 to 64 (61.9%). Nearly two in five (38.7%) women aged 40 and older in West Virginia reported not having any kind of health insurance (7).

The most recent data from the West Virginia Cancer Registry indicate that in 2002, 1242 women in the state were diagnosed with invasive breast cancer, and 268 women died from the disease. Additionally, there were 97 new cases of invasive cervical cancer and 35 deaths attributed to the disease (8).  

The West Virginia Breast and Cervical Cancer Screening Program (WVBCCSP) is part of a nationwide comprehensive public health program funded through the Centers for Disease Control and Prevention's National Breast and Cervical Cancer Early Detection Program (NBCCEDP). The goal of the program is to help uninsured and underserved women gain access to screening services for the early detection of breast and cervical cancer (9). Research has shown that through screening and early detection, breast and cervical cancer mortality rates have dramatically declined, and survival rates have improved (9,10). Although public awareness of breast and cervical cancer improves screening rates, certain populations of women still have disproportionately low screening rates (6). The WVBCCSP strives to close this health care gap by providing free or low-cost screening services to women aged 25 to 64 who are at or below 200% of the federal poverty level and who are uninsured or underinsured.

The WVBCCSP partnered with RMS Strategies, Inc (Charleston, WVa), an independent research firm, to conduct focus groups of women in three communities in West Virginia. Qualitative research methodology, such as use of focus groups, allows for interpretation of the human experience (11,12) and provides the ability to learn about breast and cervical cancer knowledge and attitudes from a sociocultural perspective. The purpose of these focus groups was to 1) obtain an understanding of attitudes and opinions about breast and cervical cancer screening tests; 2) determine motivational factors that would encourage low-income women to be screened for breast and cervical cancer; and 3) evaluate selected breast and cervical cancer educational materials according to perceived effectiveness by women.

## Methods

### Participants

Six focus groups were conducted among women aged 25 to 64 years in three communities of West Virginia — Beckley (south), Charleston (central), and Morgantown (north). These communities were selected based on the high percentage of low-income households, high percentage of uninsured or underinsured individuals, and low rates of breast and cervical cancer screening.

Sixty-nine women participated. Participants included three groups of women aged 25 to 49 years (n = 33) and three groups of women aged 50 to 64 years (n = 36). Participants were recruited randomly by telephone. The project used a random-digit–dialed sample from GENESYS (Marketing Systems Group, Fort Washington, Pa). It included the location (federal information processing standards code) and telephone numbers of households in the three study communities. The random sampling was managed by the computer-assisted telephone interviewing (CATI) system.

Eligibility requirements for participation were age, lack of health insurance, and low income. Low income was defined as earning less than 200% of the federal poverty level based on size of household (13).

### Focus groups

Focus groups were led by a professionally trained facilitator with expertise in conducting cancer research groups among Appalachian women. Each session lasted 2 hours and included 10 to 12 participants. Participants received $50 for participating and signed a consent form before the focus groups began. Each session was video and audio recorded, and the facilitator took additional notes. WVBCCSP staff members observed the sessions from an adjacent room.

The facilitator used a discussion and interview guide that was developed by the research team to lead the participants through a discussion of breast and cervical cancer attitudes and behaviors and best methods for educating low-income women about the WVBCCSP (Appendix A). At the beginning of each session, the facilitator provided a brief introduction explaining the objectives of the study, that the discussions were being recorded, and that several colleagues were observing the discussion from an adjacent room. Participants were told that there were no right or wrong answers. After the participants introduced themselves to the group, the facilitator explored women's health care issues, breast and cervical cancer awareness and knowledge, benefits of and barriers to screening, and factors that motivate women to be screened. The facilitator also led an evaluation of educational materials.

Participants reviewed four brochures. One brochure was from New Mexico Breast & Cervical Cancer Early Detection Program (14), one was from the Jefferson County (Colorado) Department of Health & Environment, and two were from the WVBCCSP. Three of the four brochures were currently being used to increase awareness and knowledge of breast and cervical cancer screening programs. The fourth brochure had recently been developed by the WVBCCSP.

Participants were asked to complete a questionnaire about the educational materials after the group discussion (Appendix B). The questionnaire asked participants to evaluate each brochure based on ease of understanding, type of information provided, the effectiveness of its encouragement to be screened for breast or cervical cancer, and the overall layout and design of the brochure.

Qualitative methods were used to analyze data (15). The facilitator studied the transcripts by age group and location along with verbal and handwritten responses to the questionnaire evaluating educational materials.

## Results

### Concerns, perceptions, and beliefs

Cost of health care and lack of health insurance were the dominant concerns among these women. These two issues kept women from routinely seeing a health care provider. A majority of the participants lacked the financial means to purchase health insurance for themselves or their families if their employer did not provide it: "My husband is going to retire in 2 years, and I have to think of how that is going to affect us with our medication and health care."

Older women tended to believe that they were "falling through the cracks" when it came to health insurance. Women who retired early (in their 50s), lost their jobs, or who were divorced or widowed had a difficult time paying for health care and health insurance; they were too young for Social Security and Medicare or did not qualify for Medicaid:

For me it is the affordability of the insurance. When my husband died, I had to drop our insurance because by the time I paid my health insurance, it left me with $250 per month to live on. If you are older than 55 and younger than 65, you are falling through the cracks because there is nothing out there to help you.

Cancer was a personal and emotional topic among participants. Nearly every participant had a personal story or experience of how cancer affected her life: "I almost didn't come tonight because my mother told me a half hour before I left that she has cancer in her lymph nodes and lungs." Religion was identified as a comforting factor that helped individuals and families get through trying times. Several participants were cancer survivors themselves: "Cancer concerns me because I have had it."

### Knowledge

Overall, participants were generally more knowledgeable about breast cancer than cervical cancer. However, they were able to identify the screening tests associated with both types of cancer. Women unanimously agreed that having a Pap test and a mammogram were extremely important and that the examinations could detect cancer in the early stages. Several participants mentioned that it was even more important for women who have a family history of cancer to be screened more frequently and at an earlier age: "If you are having problems or anyone in your family has had problems, then do it more often."

Women cited friends and family as their sources of information about breast and cervical cancer. As a result, several women shared misconceptions relating to breast and cervical cancer. For example, when asked about breast cancer, one woman said that it was more prevalent and harder to detect in women with large breasts and that women with breast implants were unable to have mammograms. Cervical cancer misconceptions included tampons causing cervical cancer and women who have had a hysterectomy, regardless of reason, no longer needing Pap tests.

Knowledge about the recommended frequency of Pap tests and mammograms varied by region and age. Women in Charleston and Morgantown were more knowledgeable about the recommended frequency of Pap tests and mammograms than were women in Beckley. Younger women expressed more uncertainty as to the frequency and purpose of Pap testing than older women.

Participants reported that they did breast self-examinations; however, some reported doing it only when they remembered rather than monthly. Older women were more likely to perform breast self-examinations than younger women. Several participants mentioned that they rely on breast self-examinations and rarely have mammograms because of cost and lack of insurance: "I lost our insurance and relied on the self-exam. I went 4 years without a mammogram."

Women discussed the role that health insurance played in relation to breast and cervical cancer screening recommendations. Some women perceived that because of their lack of insurance, doctors did not recommend Pap tests or mammograms: "As much as we want to think that they just want to make us better, they know about the insurance underneath it all. I think they know they are not making a profit."

### Barriers to screening

The major barriers that prevented women from getting a Pap test or a mammogram were cost ("We just don't go because of the cost") followed by fear of getting a mammogram ("fear of finding cancer," "fear of the unknown," "fear of pain") and embarrassment during a Pap test ("How much more vulnerable can you get when you are lying on your back with your legs in stirrups?"). Other barriers mentioned include discomfort, lack of transportation, denial, and lack of time (Table).

Health care providers have the power to help women overcome some of these barriers. For example, to overcome fear and embarrassment, participants expressed a desire for health care providers to talk to them during the procedure, letting them know what they were doing and why they were doing it.

### Motivational factors

According to focus group participants, the best motivators for screening were women sharing their cancer stories and testimonials, the buddy system, health care providers, and educational campaigns focused on increasing knowledge. Several women mentioned an educational campaign similar to "Janet's story" as being an effective campaign model for the WVBCCSP. Janet's story, a smoking cessation educational campaign, is about a woman from southern West Virginia who died in her early 40s from lung cancer caused by smoking.

Nearly all women believed they would be motivated to schedule a Pap test or mammogram if a family member or friend would go with them to the appointment. They stated that this type of buddy system would help them overcome their fears and encourage them to follow through with their appointments.

Women acknowledged the important role health care providers play in motivating women to get a Pap test and a mammogram. They noted that health care providers educating them on the examination and its purpose, along with recommending the test, would motivate them to make appointments. However, several women admitted to canceling their appointments out of fear, despite their doctors' recommendations: "It depends on how serious the doctor thinks the problem may be." However, they said that if the health care provider talked to them during the procedure, it would help eliminate their fears and reduce their embarrassment. It was also discovered that women preferred a female physician over a male physician:

I had a male gynecologist for years when I was young, and there is no comparing them to a female. They are gentler and understand where you are coming from, the emotional ups and downs. They are also sensitive.

### Awareness of the WVBCCSP

A majority of women were unfamiliar with the WVBCCSP; however, they were interested in knowing more about the eligibility guidelines and available providers in their areas. Participants who knew about the program stated that they first learned about it through their health care provider or local Department of Health and Human Resources. Those aware of the WVBCCSP went on to state that they educated their friends and family about the program.

Women who lived in Charleston and Morgantown were more likely to have heard about the WVBCCSP than women living in Beckley. Familiarity with the program tended to increase with the availability of health care providers in the focus group area. Several participants expressed concern about the limited number of health care providers in their area who participated in the program and perceived doctors' biases against patients who participated in federal and state health insurance programs.

### Recommendations for educational campaign

A majority of women believed an educational campaign would increase breast and cervical cancer awareness and knowledge and use of the WVBCCSP. By creating an educational campaign to educate women about breast and cervical cancer and the WVBCCSP, participants believed that barriers could be overcome: "I think it would definitely get more women to get the exams." Participants also felt that it was important to begin educating women at a younger age: "Start in the schools. Educate young girls about the importance of Pap smears and mammograms."

The campaign's message should be consistent, strong, and serious in nature: "We'll get through to the humor after we get through the crisis." Suggested campaign messages included: "A few moments of embarrassment are not worth risking your life over" (for cervical cancer/Pap test); "We want you for LIFE"; "Stop cancer before it stops you"; "Your life may depend on reading this"; and "Don't be a cancer statistic." Women felt that knowledge was empowering: "Maybe educate girls about it when the teachers are talking to them about getting their period. Let them know that part of becoming a woman is making sure your body is taken care of." The media were viewed as a great resource for spreading the message: "I think advertising done right is the most powerful tool we have."

The messengers of the educational campaign need to accurately portray the program's target audience, West Virginia women. The message should be delivered by multiple women from across West Virginia from diverse age groups and racial and ethnic backgrounds:

I don't think the messenger should be one person. It should be many women — older, younger, and backwoods women. In this area [Beckley], you have a lot of people who are not going to respond to advertising from strangers.

The messenger would encourage women to talk to their doctors and get screened for breast and cervical cancer.

The educational campaign should also include direct mail, television, and radio in addition to a grassroots campaign. Radio and television educational campaigns did not necessarily mean advertisements, but rather a combination of news stories and informational pieces to educate women about breast and cervical cancer and the WVBCCSP. Television and radio news stories or informational pieces should be adequately promoted so women know to tune in. Direct mail campaigns should be colorful, bold or "something of interest." The likelihood of reading direct mail varied by age and location. Younger women were more likely to read all of their mail than older women. At the same time, women in the Beckley area were more likely to read their mail than women in Morgantown and Charleston.

Information women would like included on a brochure or in an advertisement included: WVBCCSP eligibility guidelines, the importance of screening, cost of screening, and breast and cervical cancer statistics. Additionally, participants indicated that it would be beneficial to have a Web site or telephone number available to contact program staff about participating providers in their area and gain more information about the free or low-cost procedures offered.

Educating health care providers about the WVBCCSP should be an area of focus for the campaign. Women tended to rely on their doctor or clinician to recommend routine Pap tests and mammograms. Providers should also be educated about the important role they play as motivators.

## Discussion

The focus groups enabled us to meet the project's objectives. Findings from the focus groups suggest that more needs to be done in West Virginia to adequately educate women about breast and cervical cancer and programs that provide free or low-cost breast and cervical cancer screening services for uninsured, low-income women.

As expected, this population of Appalachian women was most concerned about the cost of health care and lack of health insurance. Major barriers identified through this project (i.e., cost, fear, and embarrassment) were also not surprising, given the cultural identity of the area. The lack of health insurance is a major obstacle for women and should be addressed to increase their use of screening services, such as those provided by the WVBCCSP. Our findings seem to coincide with the findings of several national surveys which suggest that poor, less educated, and uninsured populations are more likely to underuse breast and cervical cancer screening services (16).

Despite the fact that programs exist to assist low-income, uninsured, or underinsured women in receiving breast and cervical cancer screening services, many women lack knowledge and awareness of them. A primary goal of the WVBCCSP should be to increase program recognition throughout the state. To accomplish this goal, a community-based, culturally sensitive educational campaign must be developed to help women learn about the program and thereby increase breast and cervical cancer screening rates among this population. These types of campaigns have been proven to be effective in increasing screening rates among other populations of rural women, such as African Americans and Hispanics (6,17).

Public health programs and health care providers need to openly communicate with each other about the population that they serve. The WVBCCSP communicated the findings of this project to its health care providers, particularly the information about women viewing health care providers as motivators and suggested techniques that could be used during tests to help reduce the patient's fear and ease her embarrassment. This type of information exchange could help promote a more comfortable atmosphere for the patient.

The WVBCCSP has reviewed program brochures and begun updating them based on focus group findings. The general program brochure has been redesigned to include the information that the focus groups indicated they would like to see in a brochure. The program will continue to review its educational materials to evaluate whether the information accurately reflects the needs of its targeted population. Breast and cervical cancer efforts that use the words, knowledge, and suggestions of the women they serve are more likely to be effective and have a larger impact.

There were limitations to this project. Because women were recruited for this study by telephone, the opinions expressed in the focus groups may not reflect the opinions of some of the poorest residents of the state who are most likely to not have telephones. As is possible with all focus group discussions, dominant personalities may have intimidated shyer participants from fully engaging in the discussion. It is also probable that some discussion may have been inhibited because of the sensitive nature of the subject.

## Figures and Tables

**Table:** Perceived Barriers to Receiving a Mammogram and Papanicolaou (Pap) Test Among Low-Income, Uninsured, Female Focus Group Participants Aged 25 To 64 Years (N = 69) Living in Beckley, Charleston, and Morgantown, WVa, June 2005

**Mammogram Barriers**	**Pap Test Barriers**
Cost	Cost
Fear	Embarrassment
Time	Discomfort
Pain	Cold room, environment
Denial	Fear
Embarrassment	Transportation
Fear that something will be found	Time off work
Transportation	Modesty
Health insurance	Fear that something will be found
Time off work	Lack of knowledge
Modesty	Scheduling an appointment
Lack of knowledge	Hygiene issues
Husbands preventing wives from going	Waiting for results
Availability of facilities	
Unpleasantness	

## Appendices

### Appendix A. Breast and Cervical Cancer Focus Group Scripts

#### Attitudes, awareness and knowledge about breast and cervical cancer

- What concerns women in your area about their health? 
- What are some things that come to mind when I mention the word *cancer*?
- What types of regular checkups or exams are recommended for women to prevent diseases and safeguard their health? 
- How important is it for women to get a gynecological exam? 

*Probe: *frequency of exam, prevention measures

- What do you know about cervical cancer?
- How important is it for women to get a mammogram or breast exam? 

*Probe:* frequency of exam, baseline measurement, prevention measures

- What do you know about breast cancer?

#### Benefits of and barriers to breast and cervical screening

- What are the benefits of women getting a mammogram? 
- What are the benefits of women getting screened for cervical cancer? 
- What are the challenges or barriers in getting women to see and talk to a doctor or physician and get a breast exam/mammogram? 

*Probe:* problems, behavior, perceptions

- What are the barriers to getting women to see and talk to a doctor or physician and get a cervical cancer screening exam? 

*Probe:* problems, behavior, perceptions

#### Motivational factors for women

- What would motivate women to talk to their doctor or physician about getting a mammogram or cervical cancer screening exam?  
- How can a program use these motivational factors to encourage women to get a mammogram or get screened for cervical cancer?
- Who (organizations or individuals) needs to be involved in encouraging and motivating women to get a mammogram or screened for cervical cancer?
- How can these motivational factors overcome the challenges or barriers we discussed previously?

#### Program awareness and enrollment

- Do you know of any state programs available to women in your area that help pay for gynecological and/or mammogram exams? 
- How much have you heard about the West Virginia Breast and Cervical Cancer Screening Program?

*Probe:* perception of program, interest in learning more

- What is the best way for the West Virginia Breast and Cervical Cancer Screening Program to communicate with women who are eligible to participate but are not currently enrolled?

#### Educational materials

- What media outlets are the best to reach women in your area about the importance of getting screened for breast and cervical cancer? 
- If an organization were putting together educational materials (brochures, posters, etc) for a public awareness campaign to encourage women to talk with their doctor about breast and cervical cancer screening exams, what type of information should be included in the materials?
- What type of message do you think will encourage women (you) to talk to their health care professional about mammograms?
- Now, thinking about cervical cancer, what type of message do you think will encourage women (you) to talk to their health care professional about cervical cancer screening exams? 
- What type of message do you think will encourage women (you) to talk to their health care professional about cervical cancer?
- What type of photo or graphic would you like to see with your message to motivate women to get mammogram and/or cervical cancer screening exam?
- Who do you have the most trust and confidence in to deliver a message about the importance of getting a mammogram? And, for cervical cancer? 

### Appendix B. Questionnaire for Focus Group Participants About Breast and Cervical Cancer Educational Materials

#### Testing Brochures/Educational Materials

[In reviewing the brochures and educational materials, please think about the overall design of the brochures, the messages, the graphics or photos on the brochures, and the information provided.]

Evaluating messages:

- Which message will increase awareness about the WVBCCSP?
- Which message will be the most effective in encouraging women (you) to talk to their doctor/health care professional about mammogram?
- Which message will be the most effective in encouraging women (you) to talk to their doctor/health care professional about cervical cancer? 
- Which graphic or photo did you like best? Which did you like least?
- Which brochure contained the best information? Which contained the least information?
